# Electrophysiological approaches in the study of cognitive development outside the lab

**DOI:** 10.1371/journal.pone.0206983

**Published:** 2018-11-26

**Authors:** Marcos L. Pietto, Mathias Gatti, Federico Raimondo, Sebastián J. Lipina, Juan E. Kamienkowski

**Affiliations:** 1 Unidad de Neurobiología Aplicada (UNA, CEMIC-CONICET), Ciudad Autónoma de Buenos Aires, Argentina; 2 Laboratorio de Inteligencia Artificial Aplicada (Instituto de Ciencias de la Computación, FCEyN-UBA, CONICET), Ciudad Autónoma de Buenos Aires, Argentina; 3 Departamento de Computación (FCEyN-UBA, CONICET), Ciudad Autónoma de Buenos Aires, Argentina; 4 Institut du Cerveau et de la Moelle épinière, Paris, France; 5 Sorbonne Universités, UPMC Université Paris 06, Faculté de Médecine Pitié-Salpêtrière, Paris, France; 6 Coma Science Group, University and University Hospital of Liège, Liège, Belgium; 7 Departamento de Física (FCEyN-UBA, CONICET), Ciudad Autónoma de Buenos Aires, Argentina; University of Pennsylvania, UNITED STATES

## Abstract

The use of human neuroimaging technology provides knowledge about several emotional and cognitive processes at the neural level of organization. In particular, electroencephalographic (EEG) techniques allow researchers to explore high-temporal resolution of the neural activity that underlie the dynamics of cognitive processes. Although EEG research has been mostly applied in laboratory settings, recently a low-cost, portable EEG apparatus was released, which allows exploration of different emotional and cognitive processes during every-day activities. We compared a wide range of EEG measures using both a low-cost portable and a high-quality laboratory system. EEG recordings were done with both systems while participants performed an active task (Go/NoGo) and during their resting-state. Results showed similar waveforms in terms of morphology and amplitude of the ERPs, and comparable effects between conditions of the applied Go/NoGo paradigm. In addition, the contribution of each frequency to the entire EEG was not significantly different during resting-state, and fluctuations in amplitude of oscillations showed long-range temporal correlations. These results showed that low-cost, portable EEG technology can provide an alternative of enough quality for measuring brain activity outside a laboratory setting, which could contribute to the study of different populations in more ecological contexts.

## Introduction

For decades, research in cognitive psychology and neuroscience has availed itself of increasingly more sophisticated neuroimaging investigation techniques to infer how emotional and cognitive functions (e.g., perception, memory, language, emotions, and behavior) are supported or implemented by neural activity. Among them, the EEG level of analysis has the advantage of measuring neural activity directly and capturing cognitive dynamics in the time frame in which cognition occurs [1]. Due to its high temporal resolution, it allows us to explore neural mechanisms at each stage of information processing. Thus, EEG measurements have important implications in testing many hypotheses in psychology and neurophysiology. However, the inclusion of neural recordings often imposes limitations for the use of the technology outside the laboratory, because of the added burden of noise and logistics. Thus, EEG research is not applied generally beyond laboratory settings, such as in homes, schools, or on the street. Some populations, such as children and adults with cognitive disorders, are limited in attending laboratory EEG sessions, which can be lengthy and uncomfortable. Therefore, efforts that are aimed at transferring laboratory methodologies to different everyday contexts create the possibility for their inclusion in studies with greater ecological value [2–6].

Specifically, the ecological assessment in cognitive psychology involves (a) measuring a cognitive process in a context other than the laboratory (b) and measuring tasks or activities in a real-world setting [7–12]. During the last decade, different low-cost, portable EEG (LC EEG) technologies have become available. These EEG systems usually comprise a smaller array of electrodes, smaller sampling rates, the possibility of transferring the data wirelessly, and they require little adjustment to set up the recordings. However, this technology typically provides a poorer quality signal than standard research devices [13–15]. Thus, robust paradigms and new analytic methods are needed to deal with these issues. Among the first steps towards the implementation of an EEG paradigm that includes the analysis of the neural level of organization in ecological settings is the evaluation of the capacity of LC EEGs to generate reliable markers for brain oscillations that are associated traditionally with robust cognitive paradigms.

Previous studies have tested the validity of LC EEG systems, which provide evidence that supports the capacity to measure robust neural activity that is modulated by tasks [16–17] and, in particular, oddball paradigms [13–15; 18–21]. Notwithstanding, some of them pointed out limitations of the LC EEGs; for instance, they showed that the Mismatch Negativity (MMN) waveform was noisier, and it was only similar to the High-Quality (HQ) research EEGs for participants with cleaner MMN waveforms [13, 14, 21]. In sum, researchers who have investigated the quality of EEG recordings that were obtained with LC EEG systems suggested that they could be valid tools for measuring event-related potentials (ERPs) that were associated with visual and auditory oddball paradigms. However, there are other important EEG measures that are used typically in research contexts that have not been explored yet, such as modulations of the EEG frequency spectrum [22, 23] or the temporal correlations of the signal [24, 25]. These techniques are important because they allow the measurement of the distribution of neural oscillations and the dynamics of their spatiotemporal relationships. Furthermore, neural oscillations are global and crucial neurophysiological mechanisms that support many aspects of brain functioning.

Therefore, those initial validations should be extended through the application of other tasks and measures. Thus, the aim of our study was to explore, through different tasks and EEG measures, the possibility of generating robust electrophysiological markers that are able to be implemented by portable EEG technology in a school setting. Specifically, we aimed to compare the measures of LC and HQ EEG systems directly, while participants performed active (Go/NoGo) and passive (eyes-closed and eyes-open resting-state) tasks.

## Materials and methods

### Participants

Twenty-two participants (13 females, 9 males; 19 right-handedness; years of education: M = 15; SD = 3) 19–46 years old (M = 29; SD = 7) from the city of Buenos Aires were involved in this study. Participants were recruited from the School of Exact and Natural Sciences of University of Buenos Aires, and from University Torcuato Di Tella. Individuals had normal or corrected-to-normal vision and no history of psychiatric or neurological diseases. All participants provided written informed consent in agreement with the Helsinki declaration, and they were reimbursed monetarily for their participation after the two sessions of the study. All the experiments described in this paper were reviewed and approved by the ethics comittee: ‘‘Comité de Ética del Centro de Educación Médica e Investigaciones Clínicas ‘‘Norberto Quirno” (CEMIC)” and qualified by the Department of Health and Human Services (HHS, USA): IRb00001745—IORG 0001315 (Protocol 435 “Complejidad y dinámica de procesos cognitivos”).

### General procedure

Brain activity from participants was recorded with HQ EEG and LC EEG systems that were performed in two different sessions in random order and separated by ≥1 week. The difference between sessions in the time of day was around 1 hour 45 min (SD = 2 hours, 10 min). On average, HQ recording were 11min before than LQ. During each session, participants performed a Go/NoGo task and two Resting-State tasks under different conditions: with eyes-open and with eyes-closed.

Each participant was seated comfortably in a chair at 50 cm from a computer screen. The experiments were performed in an electrically shielded, dimly lit room. The preparation of the EEG head cap and electrodes took approximately 30 min for the HQ system and 5–10 min for the LC system. Stimuli were presented on a CRT monitor at a screen resolution of 1,024 × 768 pixels with a refresh rate of 60Hz, and responses were collected with a standard keyboard. All stimuli were generated using PsychoPy toolbox (v3.0) [26] for Python programming language [27] (v2.7, Python Software Foundation, https://www.python.org/).

### Go/NoGo task: Stimuli and procedures

The Go/NoGo task involves inhibitory control processes that tapped the ability to suppress pre-potent or automatic responses [28]. These processes have been associated with a brain circuit named Executive Network, which is involved in the regulation of thoughts, feelings, and behavior [29]. The stimuli were pairs of pictures of the *Pacman* game, Pacman (“Go” stimulus) and ghosts (“NoGo” stimulus), and they were created using five colors for the bodies (RGB values; *Pacman*: Yellow = 253 217 47; Ghosts: Blue = 47 140 253; Green = 48 253 72; Orange = 253 135 48; and Magenta = 250 47 253), and two colors for the eyes (RGB values; *Pacman*: Black = 36 36 13; and Ghosts: Blue = 13 66 136). Stimuli were presented in the center of the screen that occupied a visual angle of 8.84 vertically and horizontally on a gray background (RGB values, Gray = 150 150 150).

Every trial was initiated with the presentation of the stimulus that was displayed for 200 msec. Then, the stimulus was replaced immediately by a black arrow (2°) that was presented at the center of the screen for 800 msec. In each trial, the stimulus was either a *Pacman* (70% of the trials) or a Ghost (30% of the trials). Participants were instructed to respond (press the “space” button) when the *Pacman* was presented (Go trial) and to not respond when the Ghost was presented (NoGo trial). The task duration was 20 min approximately, in which each participant completed nine blocks of 90 trials (810 trials in total).

### Resting state: Stimuli and procedures

Participants were involved in two resting-state conditions. In one case, they had to remain seated and awake with their eyes closed (*eyes-closed* condition) and in the other they watched videos (*eyes-open* condition) for 5 min and 7 min, respectively. The rationale behind watching videos during the eyes-open condition was that the tasks were prepared primarily for children, in whom the resting-state was usually recorded with some background task like this to prevent large movements or from falling asleep. The participants watched one out of five videos of the *Masha and the Bear* animated television series. The episodes were chosen randomly and were voiceless.

### Semantic task

The stimuli were 72 black and white images of several types of objects (clothing, furnitures, home appliances and animals). They were displayed in the center of the screen in a light gray (RGB values, Gray = 240 240 240) background. Each trial was composed of two images from the available set, each of them was displayed for 800 msec and replaced by a gray screen for 200 msec (ISI). Participants were instructed to respond whether the stimuli were associated or not (pressing the “left” or “right” button). Each participant performed 108 trials, organized into 6 blocks of 18 trials each. The task duration was 20 min. approximately. Originally there were three conditions of different levels of semantic association between the pair of stimulus, but this manipulation was not inspected in the present study, because early visual potential were enough for the complimentary latency analysis.

### EEG recordings

Low-cost EEG recordings were collected using Emotiv EPOC+. The sampling rate was 128 Hz, and signals were bandpass-filtered between 0.5 Hz (high pass) and 40 Hz (low pass). This system has 14 electrodes placed in locations consistent with the 10–20 montage. Impedance was kept according to EPOC calibration at the threshold between YELLOW and GREEN. The presentation of the stimuli and the recording ran on the same computer. Recordings were released from the same python program using our own functions. Events were also marked using those functions. Briefly, to establish the communication between the display/data acquisition computer and the Emotiv system we used the research edition SDK (Standard Development Kit) provided by Emotiv, which allows raw data access in real time. Because the SDK was written originally for C++, we developed a binding for Python to use it with PsychoPy. The pipeline consisted of receiving the electrode signals, adding referential marks about experiments’ stimuli and responses and, finally, saving it for later analysis. The code of the experiments and the communication between the EMOTIV and the computer are available at https://github.com/mathigatti/Emotiv-Experiments.

High-quality (HQ) EEG recordings were obtained using a Biosemi 128-channel Active Two system (Amsterdam, NLD). The sampling rate was set to 512 Hz, and signals were bandpass-filtered between 0.5 Hz (high pass) and 40 Hz (low pass). The EOG signals were collected with four electrodes that were located on the outer canthi of both eyes (HEOR and HEOL) and above and below the left eye (VEOL and VEOG). The reference electrodes were placed in the right and left ear lobes and mastoids. The data were re-referenced offline to the algebraic average of the left and right mastoids. Then, the sampling rate and the electrodes of the HQ EEG were reduced according to the LC EEG system. In particular, the multidimensional data were downsampled to 128 Hz and 14 channels. The selected channels were placed on sites AF3, F7, F3, FC5, T7, P7, O1, O2, P8, T8, FC6, F4, F8, and AF4 from the 10–20 system, which was similar to the LC EEG system. The codes for the experiments were the same, but they had flags to manage how it started/stopped recording or sending marks.

### EEG preprocessing and data analyses

As explained in the previous section, both HQ and LC EEG systems ended up with a sampling rate of 128Hz and 14 electrodes after an initial preprocessing. The following steps of the pre-analysis were implemented in parallel for both systems and both tasks. Analyses were performed using EEGLAB (version 13.5.4b) [30] in MATLAB (version R2013a).

The EEG activity was re-referenced to the average reference. Removal of noisy intervals by visual inspection of the data was followed by Independent Components Analysis (ICA) using the *InfoMax* algorithm that was implemented by the EEGLAB group [30] to identify blink and saccade components in the continuous EEG recordings and to remove them from the data.

Further analyses were done specifically for each task, but in parallel for both systems.

### Event-related activity

Continuous signals were segmented into 1000 msec epochs, between 200 msec before and 800 msec after the onset of the stimulus. Baseline activity, which was defined as the mean activity in the interval [-200 msec, 0 msec], was subtracted from each epoch. Epochs that contained artifacts that exceeded a threshold of +/− 100 μV (Go/NoGo) and +/− 80 μV (Semantic) were removed automatically. Additionally, segments with residual artifacts were removed manually from the data set. Only trials with correct responses were considered for analysis. This procedure left an average of 512±52 and 186±25 trials per participant for Go and NoGo conditions, respectively, and 217±10 trials for the Semantic task. ERP waveforms were re-referenced offline to the algebraic average of the P7 and P8 channels (the closest electrodes to the right and left mastoids). Finally, separate ERP average waveforms were computed for each condition (i.e., Go vs NoGo).

To test the latency difference in ERP data, we compared the time to the peak timing of early ERP components: N1, P1, and N2 on the frontal scalp sites (F3 and F4 electrodes). Because the waveforms were shifted in time, the time-windows were defined for each system. The N1 and P1 components were defined as the negative peak in the [Go/NoGo—HQ: [80 150]; LQ: [175 245]; Semantic–HQ: [100 170]; LQ: [170 250]] msec window and the positive peak in the [HQ: [130 200]; LQ: [240 310]; Semantic–HQ: [160 230]; LQ: [240 310]] msec window, respectively, for the average ERP response in both conditions together. The N2 components were defined as the negative peak in the [HQ: [200 300]; LQ: [290 390]] msec window for the subtraction of the NoGo–Go average ERP responses. The peaks for each participant were measured by automatic selection of the EEGLAB function “findpeaks.m” (MATLAB and Statistics Toolbox Release 2013a) within those time-windows. The latencies were defined initially as the time from the onset of stimulus to the peak of interest, and the amplitudes were defined from the baseline.

To determine whether the overall waveform between both systems was conserved, the relationship in latency among ERP components was inspected. The difference between the N1 and N2 latencies and P1 latency were computed (P1-N1 and N2-P1, respectively). The rationale behind this analysis was that the temporal differences among EEG systems were not due to the quality of the signal recorded, but to the communication protocol between the EMOTIV headset itself and the computer. Statistical significance was assessed by performing Wilcoxon Signed Rank Tests (WSRT) and Mann–Whitney U test for Go/NoGo and Semantic tasks respectively.

Finally, to identify significant between-group differences across the two conditions (Go vs. NoGo), we used a combination of the Monte Carlo test and non-parametric bootstrapping by running 1.000 permutations [31]. Previous ERP studies using Go/NoGo tasks found larger amplitudes of the N2 component on frontal sites for successful responses to NoGo trials compared to GO trials, which reflected response inhibition [28, 32, 33]. Thus, contrasts were carried out independently in N2 time-windows for each EEG system (HQ: 200–300 msec, and LC: 300–380 msec) over a ROI located on frontal scalp sites (F3 and F4 electrodes).

#### Correction of single-trial latencies

A denoising algorithm that uses a wavelet decomposition of the single-trial waveform was used to obtain clean, single-trial ERPs [34–36]. They were reconstructed using only the wavelet coefficients that were related to the evoked responses, which were identified automatically using four scales and the NZT algorithm proposed by Ahmadi and Quian Quiroga [35]. The set of wavelet coefficients selected to denoise the single-trial waveforms was kept constant for all conditions and participants. As shown previously, this method improves the estimation of the single-trial ERPs significantly compared with the non-denoised, single-trial waveforms. The single-trial P1 responses were identified as the local maxima in the [Go/NoGo—HQ: [130 200]; LC: [240 310]; Language: HQ: [100 230]; LQ: [240 310]] msec time window. Then, single-trial waveforms were shifted by the latency of the corresponding P1 latency, which aligned the single-trial waveforms by the P1 peak. Finally, time-corrected ERPs (tcERPs) were estimated by averaging across trials for each participant and condition.

### Spontaneous activity

Fourteen and 16 participants completed both sessions of eyes-open and eyes-closed conditions, respectively. Therefore, the analyses were conducted only on that sample. Then, visual artifact screening procedures resulted in the exclusion of three participants for each resting-state condition. Thus, the final sample included 11 participants for eyes-open and 13 participants for eyes-closed.

#### Power spectrum

Frequency decomposition was conducted with Fast Fourier Transform (FFT) [37] in the 0.5–30 Hz frequency range for each electrode and participant in each EEG system. The power was averaged within three ROIs of four electrodes each: (1) anterior: AF3, AF4, F3, and F4; (2) central: FC5, FC6, T7, and T8 (3) and posterior: P7, P8, O1, and O2. Frequency bands of spontaneous oscillatory activity were defined on the basis of previous literature [1, 38–41]. Absolute power of the salient peaks of the spectra and relative power were calculated respectively for alpha (8–12 Hz) and beta (15–30 Hz) frequency ranges. The amplitude of each peak for each participant was obtained using automatic selection of the peaks with the function named “*findpeaks*.*m”* that was implemented in EEGLAB [30]. Relative power was computed by adding the power within the frequency band that was normalized by the power of the complete interval considered for the analysis [0.5–30 hz] (“spectopo.m”) [30].

Finally, we compared the peaks and relative power from the four ROIs using WSRT and a Holm-Bonferroni corrected p-value for multiple comparisons (p < .05). Such contrasts were carried out independently across EEG systems (HQ and LC) and resting conditions.

#### Temporal correlations

Spontaneous brain activity from each condition and EEG system was bandpass-filtered between 8 Hz (high pass) and 12 Hz (low pass) (alpha band). Then, the amplitude envelope of alpha band oscillations was calculated using the Hilbert Transform, and it was evaluated subsequently for the presence of temporal correlations using Detrended Fluctuation Analysis (DFA) [24], as described by Linkenkaer-Hansen and colleagues [25].

## Results

### Amplitudes, latencies, and effects of activity that were related to the Go/NoGo task

ERP waveform measures for the Go/NoGo task were extracted from HQ and LC systems. Specifically, amplitudes and latency peaks of N1, P1, and N2 over the frontal scalp ROI (F3/F4) were compared between both systems. The mean values of ERP latencies were used for the analysis of comparisons between EEG systems through the implementation of the WSRT. *Post-hoc* pair-wise comparisons with Holm-Bonferroni adjustments were carried out for all significant differences. The results showed that latencies were 95 ms larger with the LC system than with the HQ system (N1: z = 3.83, p < 0.01; P1: z = 4.12, p < 0.01; N2: z = 4.12, p < 0.01), but they were constant for the different peaks (N1: HQ = (109±14) ms, LC = (203±10) ms; P1: HQ = (164±15) ms, LC = (261±10) ms; N2: HQ = (256±30) ms, LC = (351±15) ms).

**Fig 1:**
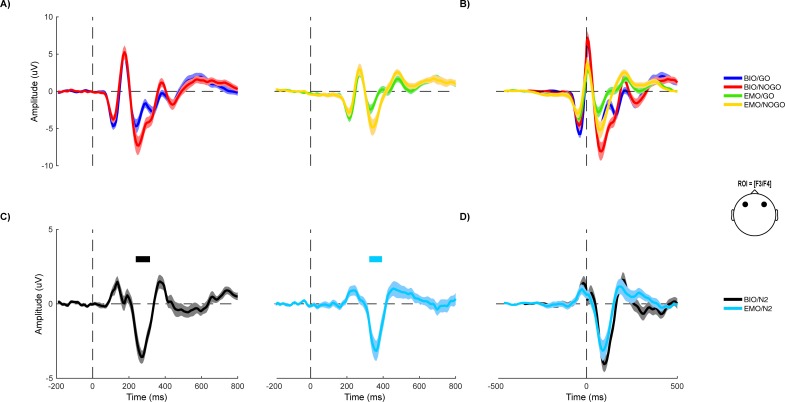
ERPs from Go/NoGo task exhibited similar waveforms in morphology and amplitude, measured with both HQ and LC systems for the selected ROI over the frontal scalp region (F3/F4). HQ and LC data included (a) ERP responses and (b) time-corrected ERP waveforms of Go and NoGo conditions in the epoch ranged from -200 msec to 800 msec for both HQ and LC systems, and (c) different ERP responses (N2: NoGo minus Go) and (d) time-corrected N2 component for HQ and LC systems.

Then, we predicted that the delays between the peaks that we observed with the LC system would be similar to the ones that we observed with the HQ system. If so, it could be possible to correct the signal by aligning the signal to the P1 peak. In fact, non-significant differences across EEG systems were observed comparing the latencies between P1 and N1 or N2 (P1-N1: z = -1.61, p = 0.11; N2-P1: z = 0.37, p = 0.71). These findings indicated that the latency shift that we observed between both EEG systems was constant throughout recording. Furthermore, this was confirmed with the temporal correction analysis, where the ERP waveforms of both conditions in LC EEG matched the corresponding conditions in the HQ EEG (Fig 1).

In order to discard that these results were specific for the task, we replicated the analysis of early visual potentials with another visual ERP task (S1 Fig). We compared the latency of N1 and P1 peaks over a frontal ROI (F3/F4). As we observed for the Go/NoGo task, latencies were 82 ms larger with the LC system than with the HQ system LC (N1: z = 4.16, p < 0.01; P1: z = 4.16, p < 0.01), but they were constant between peaks (N1: HQ = (132±14) ms, LC = (212±10) ms; P1: HQ = (193±16) ms, LC = (278±16) ms). Moreover, the difference between N1 and P1 peak latencies presented non-significant differences across EEG systems (P1-N1: z = 0.72, p = 0.47), as observed for the Go/NoGo task. This pattern of results indicated that the observed latency shift between both EEG systems was similar across tasks.

For Go and NoGo conditions, the signals that we obtained with both EEG systems were compared to investigate traditional between-group effects of this task. Grand Average data from both task conditions for frontal ROIs (F3/F4) were permuted by applying 1000 permutation draws, using a non-parametric bootstrap method (statcond.m function from MATLAB). There were significant differences between Go and NoGo conditions at the N2 time-window in both EEG systems over the frontal ROI (p<0.01). Closer inspection of the waveforms revealed that the above effects were driven by larger amplitudes of the N2 component in the NoGo condition relative to the Go condition. These findings indicated that we replicated the traditional NoGo > Go effect for N2.

### Power spectrum of activity in resting-state conditions

**Fig 2:**
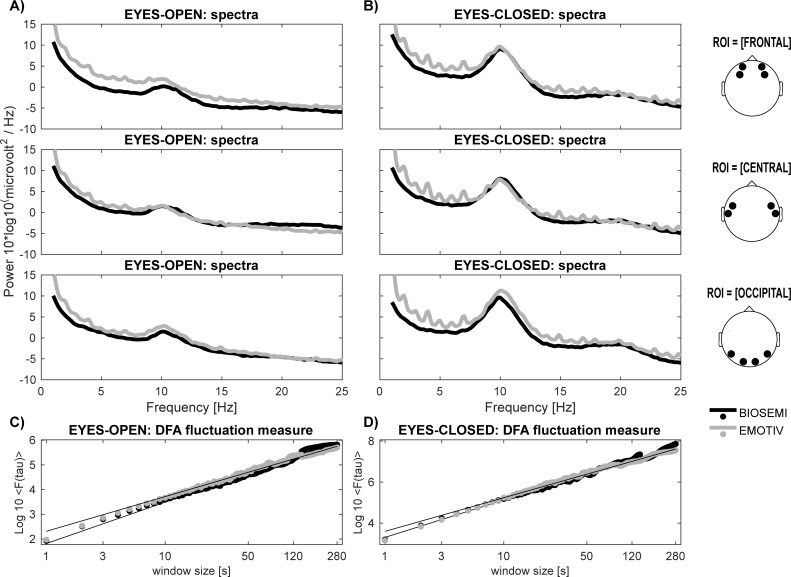
Power spectrum and DFA analysis of the resting state signals exhibited similar patterns, measured with both HQ and LC systems for the selected ROIs. (a, b) Power spectra of Biosemi (HQ) and Emotiv (LC) signals. Grand average power spectra of conditions eyes-closed (thick dashed line) and eyes-open (thick solid line) showed large peaks in the alpha frequency band (8–13 Hz) for selected ROI over frontal, central, and occipital scalp regions of Biosemi and Emotiv. **(c, d)** Double-logarithmic plots of the DFA fluctuation measure, *F (tau)*, showed power-law scaling in the time window range of 1–280 sec for both Biosemi and Emotiv data.

Power spectra during eyes-closed and eyes-open resting state conditions were compared between the LC and HQ systems. There were many possible variables? that we could use to establish a comparison between both systems, but we selected the absolute amplitude of the alpha peak and the relative power of the beta component. The main peak within the alpha frequency band (8–12 Hz) was detected automatically for each subject, condition (eyes-open and eyes-closed), system (LC and HQ), and ROI (anterior, central, and posterior) (Fig 2A and 2B). Non-significant differences were observed in the absolute median values of alpha peaks between HQ and LC systems, different conditions, and ROIs (WSRT/ eyes-open–anterior: z = -0.22, p = 0.83; central: z = 1.47, p = 0.14; posterior: z = 0.75, p = 0.46; open-closed–anterior: Z = 1.35, p = 0.18; central: z = 0.60, p = 0.55; posterior: z = -1.88, p = 0.06). In addition, the median values of relative power in the beta frequency band (15-30Hz) were not significantly different between LC and HQ systems across resting conditions and ROIs, with the sole exception of the eyes-open condition on the anterior sites (WSRT/ eyes-open–anterior: z = 2,67, p = 0.04; central: z = 1.16, p = 0.25; posterior: z = 1.79, p = 0.07; eyes-closed–anterior: z = 0.98, p = 0.33; central: z = 1.55, p = 0.12; posterior: z = 0.83, p = 0.41).

Detrended Fluctuation Analysis (DFA) has been developed for detecting long-range correlations in non-stationary time series, and it has been applied successfully to detect long-range correlations of neural oscillations in human brains with simultaneous recordings of magnetoencephalography (MEG) and EEG [25]. DFA was applied to the amplitude time series of alpha oscillations over the posterior scalp ROI (O1/O2), separately for both resting-state conditions, and for both systems. The log-log linear increase of the DFA-fluctuation parameter, *F*, started at a window size of ~1 sec and persisted until at least 280 sec in both systems (Fig 2C and 2D). The self-similarity parameter *alpha* of the DFA is the power-law exponent that characterizes the temporal correlations. Values of *alpha* between 0.5 and 1.0 indicated persistent long-range, power-law correlations. For the LC system, we observed a significant power-law scaling behavior in the eyes-closed condition in 11 out of 13 participants, and in the eyes-open condition in 10 out of 11 participants. For the HQ system, we observed a significant power-law scaling behavior in 12 out of 13 participants in the eyes-open condition, and 9 out of 11 participants in the eyes-closed condition. Average *alpha* values were obtained for LC (eyes-open = 0.59, ± 0.10 and eyes-closed = 0.67, ± 0.17; Fig 2D) and HQ (eyes-open = 0.69, ± 0.17 and eyes-closed = 0.75, ± 0.13; Fig 2C) systems. In sum, these results presented a general power-law scaling behavior that was similar between both systems (WSRT / eyes-open: z = 1.33, p = 0.18; eyes-closed: z = 1.36, p = 0.17).

## Discussion

The aim of the current study was to test the validity of a low-cost EEG system as a research tool for measuring reliable EEG data to enable researchers to explore the neurophysiological mechanisms that are associated with active cognitive and passive paradigms. The results of Go/NoGo showed that the ERPs waveforms that were associated with the Go/NoGo conditions were similar for the HQ and LC EEG systems. Moreover, for both EEG systems, the traditional NoGo > Go effect that was associated with response inhibition in the amplitude of the N2 component over frontal scalp sites was observed. These findings supported our contention that LC is suitable for measuring reliable ERPs. It is worth mentioning that the ERPs waveforms were delayed temporally for LC relative to HQ, which introduced a consistent delay in the latencies. Similar latency shifts were also observed in a motor task [14] and in other cognitive tasks, such as the Semantic task presented here or an oddball task [15]. This latency difference between the event mark, introduced directly through software, and the signal, collected through a wireless connection, has typical values of the bluetooth technology, which is that similar to the proprietary EMOTIV wireless protocol. Other studies decided to include an input event signal through a cable plugged into a couple of electrodes to solve to latency issue, losing two electrodes and the wireless capability [13–14]. Instead, we opted for an analytical approach. The analysis of the relative timing of ERP peaks revealed non-significant differences between the temporal relationships of event-locked responses from both EEG systems. Following this idea, we proposed a procedure to correct the timing offline that was based on wavelet, single-trial denoising and peak detection that removed the differences in latencies.

The results from the resting-state conditions also supported the consistency between EEG systems. First, we found non-significant differences in the analyses of the spectra that were revealed in both the peak of the alpha range and the relative power of the beta range between the two EEG systems. Second, we explored more complex measures such as DFA. Interestingly, the results indicated that spontaneous alpha oscillations of both EEG systems had significant long-range temporal correlations during resting state conditions (eyes-open and eyes-closed), with a decay that scaled as a power-law function and with a remarkably invariant scaling exponent. These findings converge with a prior study [25] that reported that spontaneous activity was characterized robustly by long-range temporal correlations that decayed as a power-law function. Interestingly, these measures were also consistent between EEG systems, thus creating the possibility to implement more complex analyses on data with these low-cost systems.

Finally, these findings are the result of joint efforts from us and others that are aimed at transferring laboratory methodologies to different contexts, such as schools or workplaces, thus creating the possibility of extending their inclusion to studies with greater ecological value. Nevertheless, in the present study, the evaluation was based on data that were acquired from a small sample of adults who performed a particular cognitive task in a laboratory setting. Future research should expand the evaluation to other contexts outside the lab. In addition, the designs should include larger sample sizes, other paradigms, and potentially different populations, such as children and patients. These approaches would pave the way to a broad spectrum of possibilities for future targeted applications of these LC EEG systems.

## Supporting information

S1 Fig Early ERPs from semantic task exhibited similar waveforms in morphology, measured with both HQ and LC systems for the selected ROI over the frontal scalp region (F3/F4). HQ and LC data included ERP responses and time-corrected ERP waveforms in the epoch ranged from -200 msec to 800 msec for both HQ and LC systems.
(EPS)
